# Dermatology Publications on COVID-19 during the First Pandemic Year: Creativity or Opportunism?

**DOI:** 10.3390/life13040953

**Published:** 2023-04-05

**Authors:** Paolo Amerio, Federica Giuliani, Marco Coppola, Fabio Lobefaro, Giulio Gualdi

**Affiliations:** Deparment of Medicine and Aging Science, UOC Dermatologia University of Chieti-Pescara, 66100 Chieti, Italy

**Keywords:** COVID-19, publication, dermatology

## Abstract

Introduction: Dermatologists had to face several challenges during the COVID-19 pandemic. In this scenario, a large amount of data has been produced and published. Objectives: We present a literature analysis of publications on COVID-19 in the dermatology field in the first year of the pandemic. Methods: The research was carried out by searching the PubMed database using keywords related to “COVID-19” combined with the keyword “Dermatology” in the “affiliation” search field and collecting articles published from February 2020 to December 2020. Results: A total of 816 publications from 57 countries were retrieved. Overall, publications increased notably along the timespan considered in this study and appeared to be closely linked to pandemic progression in different countries. In addition, article types (i.e., commentaries, case reports, original research) appeared to be strictly influenced by the pandemic’s progression. However, the number and category of these publications may raise questions regarding the scientific relevance of the messages reported. Conclusions: Our analysis provides a descriptive quantitative analysis and suggests that publications do not always respond to real scientific needs but are sometimes linked to a need/opportunity for publication.

## 1. Introduction

The coronavirus disease (COVID-19) pandemic has put unprecedented pressure on healthcare systems worldwide [1]. Extensive measures have been implemented to reduce and prevent transmission [1]. As COVID-19 has spread rapidly, the research community has been active in publishing articles on this dreadful disease. The scarcity of information, especially in the first months of the pandemic, and the necessity of data sharing prompted journal editors to establish COVID-19 repositories or paper special editions and to speed up the publication of papers on this subject [2]. The urgency to share materials also favored the publication of preliminary papers (i.e., reports that have not yet undergone peer review) on dedicated databases such as MedRixv.

Although dermatologists were not directly involved in the treatment of COVID-19 patients, they were interested in the evaluation of cutaneous consequences of infection and the implications of COVID-19 infection in dermatological patients chronically treated with immunosuppressors and skin cancer oncological therapies [3,4]. Thus, dermatologists had to thrive in the management of skin conditions, owing to the dramatic scarcity of information [5,6,7]. In this context, a large amount of material has been produced and published in the dermatological field. However, from the early stages of the pandemic until late in 2020, some authors have questioned the utility of this flood of papers with little or rapid peer review on COVID-19, and have underlined the dangers hidden in the balance between speeding up publishing [8] and eagerness for information [9]. Transparency and the risk of over-estimation of published data with multiple publications about the same dermatological cases were already of concern in the middle of 2020 [10].

Here, we present a literature analysis of publications on COVID-19 in the dermatologic field during the first year of the COVID-19 pandemic. The purpose of this review is to explore how, in a relatively short but highly critical time period, publication flows have developed concerning the pandemic trend. We focused our attention on the first year of the pandemic; after this period, in fact, these flows stabilized, and the literature “normalized.”

## 2. Material and Methods

A comprehensive search of the PubMed (URL https://pubmed.ncbi.nlm.nih.gov/ accessed 2 February 2021) database was performed from 1 February 2020 to 31 December 2020. An advanced search was performed using the search fields “title/abstract” and “affiliation.” In the first search fields, the common set of words used were: “CoViD,” “COVID-19,” “SARS-CoV-2,” “SARS-CoV,” “2019-nCoV,” “Novel Coronavirus,” and “Pandemic.” These were combined with the words “Dermatology” or “Skin” in the “affiliation” search field. Moreover, research designs were independently searched for: letter, case reports, epidemiologic studies, randomized controlled trials, and systematic reviews.

Data records retrieved from the databases were imported into Excel for further analysis. Data extraction was performed by one author (GG) and was checked by other authors (FG and MC).

Three independent investigators (PA, MC and FL) analyzed the title, abstract, and full text of each collected publication to determine eligibility based on the following inclusion criteria: (1) at least one of the authors should display an affiliation in dermatology, and (2) the subject of the paper should have been strictly related to COVID-19. The following information was recorded for articles included in this study: journal impact factor, date of publication, and country of origin of the authors. Statistical analyses were performed using R software environment for statistical computing (version 3.4.1; URL: http://www.r-project.org/ accessed 4 July 2021). Publications from the European geographical area were also categorized according to the principal topic of the publication: description of COVID-19 skin manifestation, treatment of COVID-19 with drugs used in dermatology, approach to skin disease during the COVID-19 pandemic, skin research and COVID-19, position papers and commentaries, impact on the management of dermatologic clinics, psychological impact of COVID-19, and miscellaneous topics.

## 3. Results

**Figure 1:**
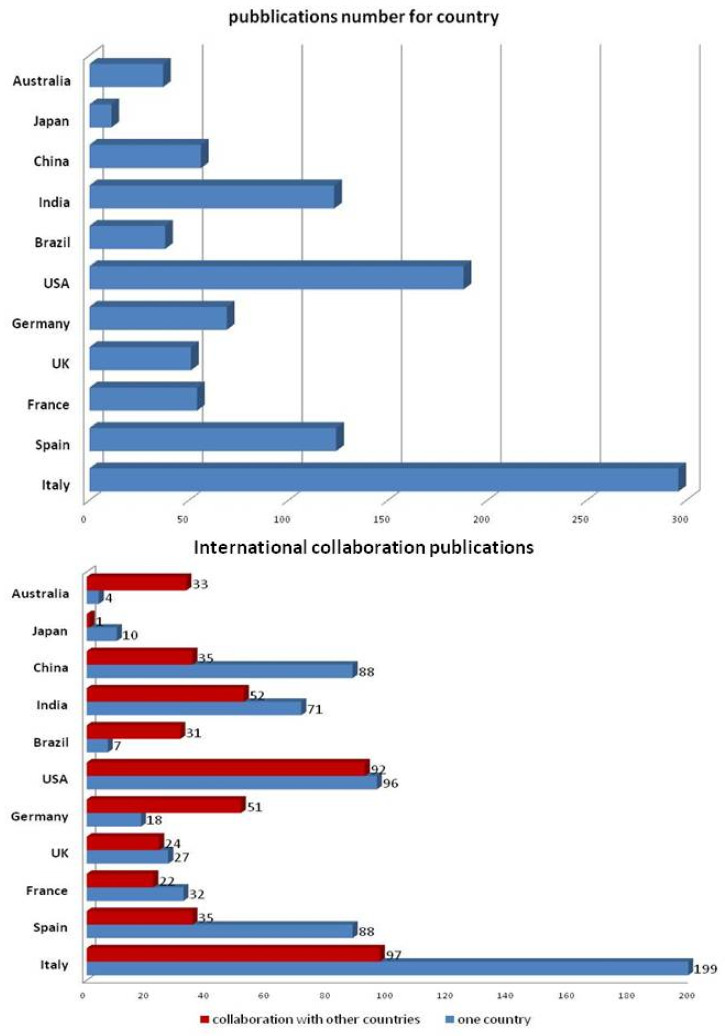
(**upper)** Indexed COVID-19 papers by country. (**Lower**) Collaboration between authors from different countries.

**Figure 2:**
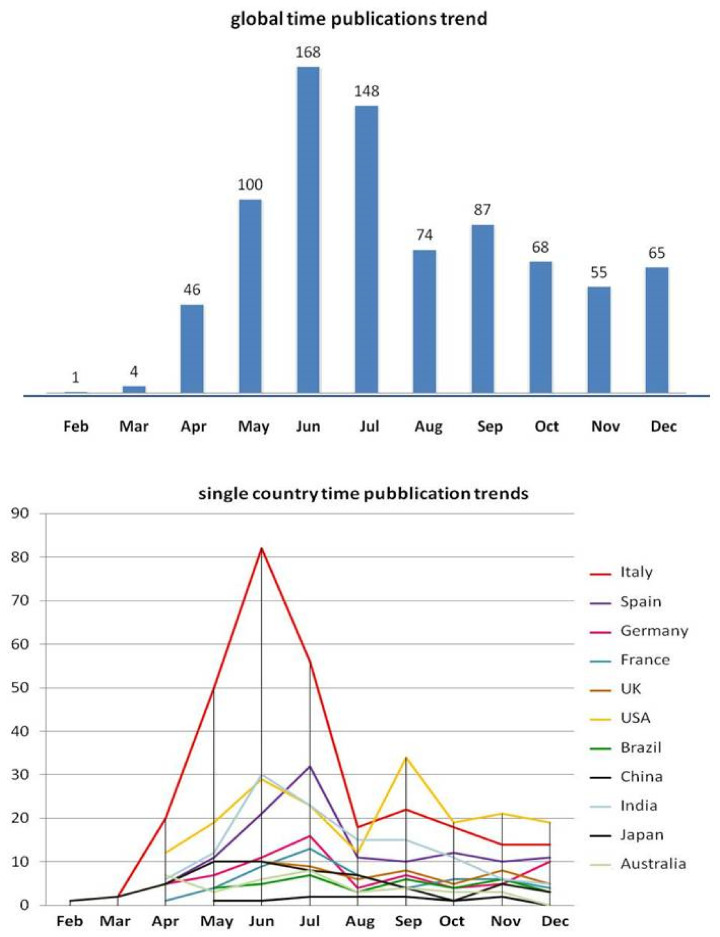
(**Upper**) Distribution of COVID-19 papers by time. (**Lower**) Distribution of COVID-19 papers by time and different countries.

**Figure 3:**
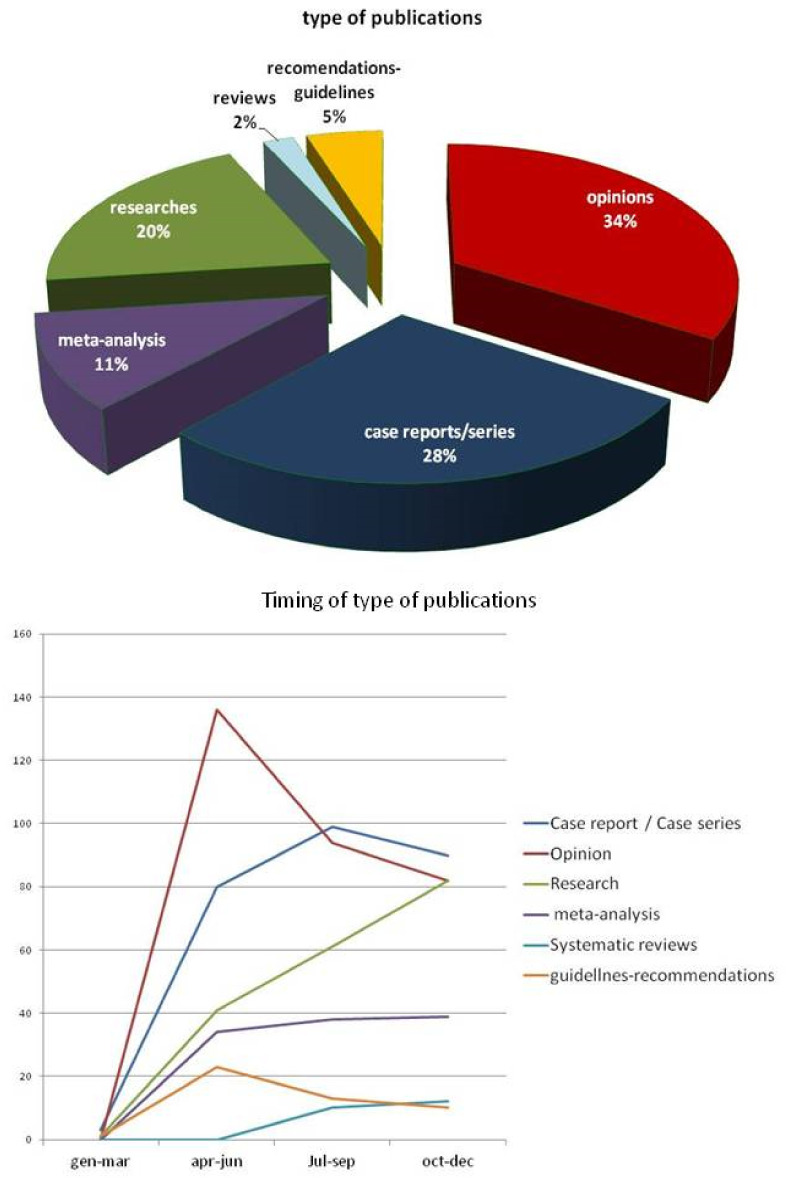
(**Upper**) Distribution of COVID-19 papers based on article type. (**Lower**) Time course and type of publication.

A total of 1523 documents were retrieved. After removing duplicates and papers failing to fulfill the inclusion criteria, the study sample was composed of 816 publications from 57 different countries. Figure 1 reports indexed COVID-19 papers by country: Italy was the most productive country with 296 publications. The United States of America ranked second (n = 188), followed by Spain and India (n = 124 and 123, respectively), Germany (n = 69), China (n = 56), France (n = 54), the United Kingdom (n = 51), Brazil (n = 38), Australia (n = 37), and Japan (n = 11). All other countries accounted for only one or two publications. The total number of papers reported in Figure 1a is higher than 816 because of the presence of collaborative research from authors of different nationalities. In fact, authors from different countries showed a distinct trend toward collaborating with peers for publications (Figure 1b), For example authors from China (39%) and authors from Italy (48%) did report at least one collaborative paper with authors from other countries. The first paper indexed on PubMed concerning COVID-19 and dermatology was published in China in February 2020. Figure 2a shows the distribution of COVID-19 papers by time, and Figure 2b combines publication times and countries. The overall trend shows a progressive increase in the number of publications until a peak was reached between June and July. Publication numbers decrease in August and September, and then stabilize at a plateau (about 60 per month) until December. Analysis of the temporal trends of publications in single nations shows very different trends. In Italy, there was first a higher peak at the beginning of summer, and then a second, lower peak in autumn. The same trend was observed in publications from other European countries. United States-based publications showed a first, lower peak in summer, followed by a second, more abundant number of publications in autumn, and a third rise in the number of publications in winter. A similar trend, with three distinct peaks in the number of publications, is also present in Brazil. Figure 3a shows the distribution based on article type: more than one third of the publications were opinions (34%), 28% were case report or case series, and 20% were research articles. The remaining categories consisted of meta-analyses (11%), guidelines/recommendations (5%), and reviews (2%). Figure 3b shows the time course and type of publication: opinion articles and guidelines/recommendations showed a peak in the first half of the pandemic, significantly decreasing in the second, while research articles increased progressively throughout the study period. The publications appeared in 125 different scientific journals, but only 47 were in the dermatology category. Seventy-six percent (n = 623) of articles were published in 12 dermatological journals. Seventy-five journals (60%) published only one article. The journals that published the largest number of articles were: *Dermatologic Therapy* (IF 2.327; n ° = 210/25.7%), the *Journal of the European Academy of Dermatology and Venereology* (IF 5.248; n ° = 128/15.7%), the *Journal of the American Academy of Dermatology* (IF 8.277; n ° = 56/6.9%), the *International Journal of Dermatology* (IF 2.067; n ° = 50/6.1%), *Clinical and Experimental Dermatology* (IF 1.977; n ° = 44/5.4%), the *British Journal of Dermatology* (IF 7.0; n ° = 36/4.4%), and the *Journal of Dermatological Treatment* (IF 2.185 n ° = 33/4%).

As for the topics of the publications, we analyzed only articles published in European countries and found that 157 were descriptions of COVID-19 skin manifestation, 30 were about the treatment of COVID-19 with drugs used in dermatology, 104 were about approaches to skin disease during the COVID-19 pandemic, 37 were about skin research and COVID-19 (mainly histopathological descriptions of skin manifestation), 131 were position papers and commentaries, 61 were about impacts on the management of dermatologic outpatient and hospital clinics, 15 were about the psychological impacts of COVID-19 in dermatological patients, and 61 were about miscellaneous topics, such as the use of teledermatology during lockdown.

## 4. Discussion

The aim of this study was to summarize and analyze the evolution of the impact of the COVID-19 pandemic on scientific production in the dermatologic field. We were able to collect a large number of publications that were classified according to country of affiliation from February to December 2020. Publications increased notably along the timespan considered in this study and appeared to be closely linked to pandemic progression in different countries. Publication start date followed the worldwide spread of the virus, starting in China (February), followed by Italy (March), and then the rest of Europe and other countries.

As reported by other publication analyses of COVID-19-related papers [10] in the dermatologic field, publication types in the first part of the pandemic year were mainly commentaries, letters to the editor, and recommendations. Since April 2020, as knowledge of the impact of COVID-19 on skin became more evident, these were promptly replaced by clinical trials, original research, and large case series papers.

In fact, initial lack of objective data and knowledge led to the rapid publication of opinion articles, recommendations, and guidelines in the first quarter of the study period in an attempt to manage fear, anxiety, and the new challenges imposed by the virus. In addition, case reports and cases/series numbers increased as authors faced different aspects of COVID-19 skin presentation and a higher number of dermatological patients were exposed to the virus. Simultaneously, the number of research articles started to grow rapidly following the possibility of collecting data and samples from a wider number of patients, followed by meta-analyses and reviews. Certainly, the emergence of the pandemic has led to a greater need for new knowledge sharing in the context of the management of dermatological patients and their treatments, new work procedures, emerging diseases induced by COVID-19, and containment measures.

However, notably, more than half of the 270 opinion articles were published between February and March, storming the dermatologic scientific community with a plethora of information at a time when not much scientifically based evidence was available. In some cases, the information given was only opinion-based; however, several papers presented useful information on skin presentation of COVID-19-related diseases [3].

There were also several papers on the opportunity for skin disease treatment modification because of COVID-19 infection, both in inflammatory conditions such as psoriasis [11] and oncologic conditions such as melanoma [12].

Many studies have described the safety of certain immunosuppressive agents used for skin diseases in COVID-19 cases, most of which were single case reports with very little evidence to support claimed safety [13], even suggesting the usefulness of concomitant dermatological treatments for COVID-19-driven inflammation [14,15]. However, during the dramatic days of the early pandemic, even this scant information could have been beneficial.

Unfortunately, however, many other papers have been inadequate, engulfing dermatologic journals with matters showing little correlation with COVID-19-associated dermatological problems. Some examples include: (1) suggesting that listening to music could be a valid alternative to dermatological consultations for chronic inflammatory dermatologic diseases [16], (2) presentation of improbable or unprovable hypotheses, such as one study that hinted at how the impact of lockdown could have lowered melanoma diagnosis due to sun avoidance during forced home segregation [17]; and (3) odd analysis of the shifting of the market strategy for cosmetics during the pandemic [18] as if it was relevant at that time when robust data on disease impact on skin were much-needed.

During the 12-month period evaluated in our review, most frequently, papers were about skin manifestation of COVID-19, with some very useful review papers that helped in recognizing COVID-19-related cases. These could be pointed to as an example of concomitant rapid and worthwhile publications [4]. The second most-frequent type of publication was commentary or position papers. Many of these were from scientific societies or scientific groups and comprised very useful position papers that gave direction in those hard times; however, a larger number were, as already mentioned, only opinions on the most variable topics that were not directly correlated with COVID-19. The next most frequent type of publication included approaches to skin disease during the pandemic. These articles covered the management of a great variety of skin conditions during the pandemic, addressing the risk and impact of SARS-CoV-2 infection in dermatological patients, including psoriasis, oncologic disease, hidradenitis suppurativa, and lymphomas [12,19,20,21]. Only 37 papers were about disease pathology research in patients with SARS-CoV-19-induced skin manifestations. These studies mainly focused on the histopathology or histochemistry of skin biopsies describing inflammatory skin changes. There were also papers on the impact of pandemic-induced lockdown on the management of dermatological outpatient clinics. Descriptions of the management of outpatient clinics or suggestions on how to deal with the pandemic were given for the following outpatient clinics: dermato-oncologic, immune-mediated diseases, sexually transmitted disease, dermo-surgical, aesthetic, tricological, and phototherapy. Although many of the suggestions were sound and practical, sometimes the information provided proved to be confusing. In the case of phototherapy, in the same month (June 2020) the following advice was given: (1) to stop phototherapy because of its immunosuppressive action that could ultimately impact the probability of being infected by SARS-CoV-19 [22], and (2) to improve the use of phototherapy because the production of vitamin D by UVB nb could somehow protect from the virus [23].

Surprisingly, given the high impact of the disease on the lives of people and the profound impact of lockdown measures on the populations of several countries, only a few papers dealt with the psychological burden on patients and doctors due to the pandemic [24].

There were also imaginative and creative hypotheses on the use of existing dermatological drugs for the treatment of COVID-19 disease: omalizumab, montelukast, hydroxicloroquine (HCQ), apremilast, doxicicline, ivermective, anti-alpha 5 reductase agents, anti-IL-17, and endothelial receptor antagonists were all proposed as treatments for COVID-19. Some of these studies, however, were at least questionable in their structure; for example, in one study [25], HCQ was suggested as a potential treatment for COVID-19 on the basis of a report of a case series of rheumatological and dermatological patients using this drug. However, these patients were never infected with COVID-19 and had never had close contact with a COVID-19 case. Thus, this paper was merely a list of patients undergoing HCQ treatment and a wishful conclusion on the role of HCQ on COVID-19.

Even more interesting was a paper on the association of regional diets with COVID-19 risk and COVID-19 symptoms [26]. This paper was however usefull to contrast the bad habit of patients to seek information on the capacity of diet to protect themselves from this disease. Finally, 61 papers were published on miscellaneous topics, the majority of which were about the utility of teledermatology consultations and virtual e-learning, but also included reports of media coverage of dermatologic conditions and dermatological-related Google searches during lockdown [27,28].

The number and category of these publications may raise some questions regarding the scientific relevance of the messages reported and the real role of the review procedure during the early pandemic period. Regarding the qualitative aspect of the publications, it should be emphasized that although about a quarter (210 papers) of the publications were published in a medium-low impact dermatological journal, another quarter (220 papers) were published in three of the top five dermatological journals.

Most papers on dermatological topics related to COVID-19 were published in three journals (*JAAD*, *JEADV*, and *Dermatological Therapy*). Each of these three journals had fast-track publication policies that allowed new information on COVID-19 to become widely acknowledged. However, it has been noted in the media and on the Internet (URL: https://twitter.com/deevybee/status/1297404282262749184 accessed 2 June 2021) that in the case of one particular journal, many articles were written by the same group of authors, raising some questions about the practices of prolific editors who publish in their own publications (PEPIOPs).

## 5. Conclusions

The purpose of our review was not to compare the clinical results on a single topic but to narrate how, in a relatively short but harshly critical period, flows of publications have developed concerning the pandemic. The collected data show a massive flow of publications fueled by the pandemic emergency in the dermatological field. As other authors [8,9,10] have pointed out, together with papers of undoubted value, many articles dealt with frivolous or uninteresting topics, making it difficult for the dermatologist to navigate this storm of information. The main limitation of this paper is its intrinsic bias because it was based on the PubMed database, and results may differ according to other databases.

## Data Availability

Not applicable.
